# Improving the Acquisition, Interpretation, and Documentation of Electrocardiograms (ECGs) in Patients Suspected of Having Acute Coronary Syndrome: A Clinical Audit Conducted in Emergency Departments Across Sudan

**DOI:** 10.7759/cureus.82892

**Published:** 2025-04-24

**Authors:** Isra Hamed Dafaalla Idrees, Sara Hafiz Mohammed Alamin, Mohammed Osman Ahmed Osman, Mazin Algassim Abdelnassir Mohamed, Nagwan Noaman Ahmed Mohammed, Wefak Abdelsamad Hassan Omer, Mohamed A Awad Elkarim, Malaz Kamaleldin Omer, Mustafa Mohamed

**Affiliations:** 1 Medicine and Surgery, Nile Valley University, Khartoum, SDN; 2 Medicine, Nile Valley University, Khartoum, SDN; 3 Internal Medicine, The National Ribat University, Khartoum, SDN; 4 Medicine, The National Ribat University, Khartoum, SDN; 5 Medicine, Ahfad University for Women, Khartoum, SDN; 6 Medicine, Alzaiem Alazhari University, Khartoum, SDN; 7 Internal Medicine, University of Gezira, Wad Madani , SDN; 8 Internal Medicine, Sudan Medical Specialization Board, Khartoum, SDN; 9 Internal Medicine, Prince Othman Digna Teaching Hospital, Port Sudan, SDN

**Keywords:** acute coronary syndrome, aha/acc guidelines, clinical audit, ecg, emergency department, sudan

## Abstract

Background: Early electrocardiogram (ECG) acquisition, interpretation, and documentation are critical in the management of patients with chest pain. The American Heart Association/American College of Cardiology (AHA/ACC) guidelines recommend that an ECG should be obtained and interpreted within 10 minutes of arrival in the emergency department (ED) for any case of acute coronary syndrome (ACS). This study aimed to assess and improve adherence to these guidelines in the Sudanese ED.

Methods: A clinical audit was conducted in two cycles at Kassala Teaching Hospital in Sudan. The first cycle evaluated baseline adherence to ECG acquisition, interpretation, and documentation times. Interventions, including educational sessions and posters, were implemented to address gaps. The second cycle assessed the impact of these interventions. Data were collected using a standardized checklist and analyzed using the Statistical Package for the Social Sciences software, version 25 (IBM Corp., Armonk, NY).

Results: In the first cycle (n = 25), the average time from arrival to ECG acquisition was 29.84 minutes, with only 4% of patients having documentation completed within 10 minutes. After intervention, the second cycle (n = 22) showed significant improvement, with 81.8% of patients achieving the 10-minute target. Documentation quality also improved, with "Rhythm," "Rate," and "ST segment" being the most frequently documented elements.

Conclusion: Targeted interventions have significantly improved adherence to AHA/ACC guidelines for ECG acquisition, interpretation, and documentation in Sudanese EDs. These findings highlight the importance of continuous education and monitoring to enhance the quality of care for patients with suspected ACS.

## Introduction

Acute coronary syndrome (ACS) remains one of the leading causes of morbidity and mortality worldwide, necessitating prompt diagnosis and treatment to improve patient outcomes. An electrocardiogram (ECG) is the primary diagnostic tool used when patients present with chest pain or related symptoms. According to guidelines set by the American Heart Association/American College of Cardiology (AHA/ACC), an ECG should be performed and interpreted within 10 minutes of a patient's arrival at the emergency department (ED) for suspected ACS cases [1]. This recommendation is supported by evidence that early ECG acquisition facilitates timely therapeutic interventions, such as thrombolysis or percutaneous coronary intervention, which are critical for preserving myocardial tissue and reducing mortality [1].

Research indicates that approximately one-third of ACS patients receive an ECG within the critical 10-minute window upon arrival in the ED. For example, a prospective study demonstrated that only approximately one-third of ACS patients had an ECG performed within this target timeframe [2]. Extended door-to-ECG times have been consistently linked to delays in treatment and, consequently, poorer clinical outcomes. More recent studies and systematic reviews have reinforced these findings, suggesting that even with improvements in ED protocols, achieving the 10-minute benchmark remains challenging. This is especially pronounced in resource-limited settings, where shortages of diagnostic equipment, delayed triage processes, overcrowding, and limited availability of skilled personnel further complicate the timely acquisition of ECGs [3].

Emerging evidence indicates that targeted strategies, such as implementing standardized triage protocols, focused staff training, and dedicated ECG documentation templates, can significantly improve adherence to the 10-minute ECG guideline. However, structural obstacles remain. In many settings, prior discussions have focused on the limited availability of catheterization laboratories during off-hours. In contrast, our findings and subsequent discussions also highlight other critical barriers in the Sudanese context: inconsistent triage practices, ED overcrowding, and insufficient training in ECG acquisition and interpretation. These factors have a direct impact on the timeliness of care and overall patient outcomes [4].

In Sudan, where healthcare resources are frequently constrained, adherence to AHA/ACC guidelines for ECG acquisition and interpretation is suboptimal. This clinical audit was conducted to evaluate and improve the efficiency and accuracy of ECG acquisition, interpretation, and documentation in the Sudanese EDs. By identifying specific local barriers, such as delayed triage processes, lack of continuous ECG technician availability, limited ECG machines, and variable levels of staff training, this study aims to implement targeted strategies that may include protocol revisions, educational interventions, and improved documentation systems. Although currently focused on immediate postintervention outcomes, these findings also underscore the need for regular reaudits to ensure sustained adherence and to validate the applicability of our interventions to similar low-resource settings.

Conducting such audits not only provides a structured framework for assessing and enhancing current clinical practices but also fosters a culture of continuous quality improvement. Regular audits and ongoing evaluations can help healthcare providers remain aligned with the evolving best practices, ultimately leading to sustained enhancements in patient care and clinical outcomes.

## Materials and methods

Study design

This study was conducted as a clinical audit in two cycles at Kassala Teaching Hospital in Sudan. The primary objective was to assess adherence to recommended ECG acquisition, interpretation, and documentation timelines in the ED. The first cycle served as a baseline assessment, identifying delays in ECG processing. Subsequently, targeted interventions, including educational sessions and informative posters, were introduced to address these gaps. The second cycle evaluated the impact of these interventions on improving adherence to recommended practices.​

This audit aimed to evaluate and enhance the timeliness and accuracy of ECG acquisition and interpretation in EDs to improve patient outcomes. In resource-limited settings, such as Sudan, implementing these audits can identify specific barriers to timely ECG processing. Addressing these challenges through targeted interventions can optimize the use of available resources, reduce delays, and improve adherence to established guidelines.​

Furthermore, regular clinical audits foster a culture of continuous quality improvement within healthcare institutions. They provide a structured framework for assessing current practices, implementing evidence-based interventions, and monitoring their effectiveness over time. This cyclical process ensures that healthcare providers remain responsive to evolving best practices, ultimately leading to sustained improvements in patient outcomes and the quality of care delivered.​

Data collection

Data were systematically collected using a standardized checklist based on the AHA and ACC guidelines [1]. The audit checklist used in the study is included in the Appendix. Table 1 includes the checklist, which includes the following key parameters.

**Table 1:** Audit parameters for ECG acquisition, interpretation, and documentation in EDs Key parameters used to assess the efficiency and documentation quality of ECG processes in the ED are outlined. It includes time intervals for different stages of ECG handling, the rationale for ECG performance, and the quality of documentation ED: emergency department; ECG: electrocardiogram Source: [1]

Parameter	Description
Time from arrival to ECG acquisition	Duration from patient arrival at the ED until the ECG is acquired
Time from ECG acquisition to interpretation	Duration from the moment the ECG is completed until a clinician interprets the results
Time from interpretation to documentation	Duration from when the ECG is interpreted until the findings are formally documented in the patient record
Indication for ECG	Reason for performing the ECG, distinguishing between a typical presentation (e.g., typical chest pain) and atypical presentations
Quality of ECG documentation	Completeness of the documented findings, including details such as rate, rhythm, axis, ST segment, QRS complex, and PR interval

Data collection was conducted by a trained team of healthcare professionals stationed throughout the ED. These individuals ensured real-time documentation and adherence to data collection protocols.

Study population and setting

This study focused on adult patients (18 years and older) presenting to the ED of Kassala Teaching Hospital in Kassala, Sudan, with chest pain or other clinical features indicative of ACS. Kassala Teaching Hospital serves a diverse patient population in a resource-constrained setting.​ Patients were excluded if they presented with chest pain attributable to trauma, were referred from other healthcare facilities, or declined ECG acquisition.​

The study population comprised a mix of male and female patients across various age groups. A significant proportion had comorbidities such as hypertension, diabetes mellitus, or a history of cardiac disease. These demographic characteristics reflect the typical patient population presenting with suspected ACS in this region.

Sampling technique

A total coverage sampling approach was employed to include every eligible adult patient with suspected ACS during the study period. The first audit cycle was conducted from March 19, 2024, to May 31, 2024 (approximately 2.5 months), and the second cycle from August 26, 2024, to December 26, 2024 (four months). These periods were selected based on logistical considerations and to capture a representative sample of patient presentations.​

The total number of patients included was 47, with 25 in the first cycle and 22 in the second. While this sample size may appear modest, it aligns with recommendations for clinical audits, where sample sizes of 20-50 cases are often sufficient to assess adherence to clinical standards and identify areas for improvement. The primary goal was to evaluate process adherence rather than to achieve statistical power for hypothesis testing (studylib.net).

The unequal durations of the audit cycles were influenced by factors such as staff availability and institutional scheduling. Despite the longer duration of the second cycle, the number of eligible patients was slightly lower, which may reflect variations in patient admission rates or seasonal factors. Importantly, the total coverage sampling method ensured that all eligible patients were included during each period, thereby maintaining the integrity and comparability of the data across both cycles.

Data and statistical analysis

Data were entered and analyzed using the Statistical Package for the Social Sciences, version 25 (IBM Corp., Armonk, NY). Descriptive statistics were used to summarize the data, with results presented as frequencies and percentages for categorical variables and means for continuous variables. To assess the significance of changes observed between the first and second audit cycles, inferential statistics were applied. The chi-square test was used to compare proportions across cycles. A p value of less than 0.05 was considered statistically significant.

Audit cycles

First Cycle: Baseline Assessment

The first cycle, conducted from March 19, 2024, to May 31, 2024, established baseline adherence to ECG acquisition, interpretation, and documentation times. The results identified significant inefficiencies, with an average time of 29.84 minutes from patient arrival to ECG acquisition. Additionally, only 4% of patients had their documentation completed within the recommended 10-minute timeframe [1]. These findings underscore critical delays in ECG processing, necessitating targeted interventions to optimize workflow efficiency.

Intervention Phase: Implementation of Targeted Strategies

From June to August 2024, we implemented a series of structured interventions to address the shortcomings identified in the initial cycle. These interventions included educational sessions, visual aids, and targeted workflow optimizations aimed at improving the performance of ED physicians and nurses.

The educational sessions were designed to emphasize the urgency of timely ECG acquisition, accurate interpretation, and thorough documentation in alignment with AHA/ACC guidelines. The curriculum focused on a systematic approach to ECG interpretation, including critical components such as heart rate and rhythm, electrical conduction, axis determination, R-wave progression, voltage assessment, and ST/T wave changes. Each session lasted 30 minutes and was delivered through a hybrid model, combining in-person and virtual formats. The structure of the sessions included didactic lectures followed by interactive discussions, allowing participants to engage with real-life case scenarios. A total of 25 ED staff members (representing 75% of the total staff) voluntarily participated in these sessions, and postsession evaluations indicated that a significant majority of participants felt their ECG interpretation skills had improved.

In addition to the educational sessions, visual aids and reminders were strategically placed within the ED. Informative posters detailing best practices and optimal ECG processing times were displayed in key areas to reinforce awareness and adherence among healthcare professionals. To evaluate the effectiveness of these visual aids, a compliance audit was conducted after installation, revealing a 20% increase in adherence to ECG guidelines.

Workflow adjustments were also implemented to minimize unnecessary delays. These included relocating ECG machines to more accessible areas within the ED, revising triage protocols to prioritize suspected ACS cases, and adjusting staffing levels during peak hours. These changes aimed to ensure rapid assessment and ECG acquisition for suspected ACS cases, ultimately streamlining operations and improving patient care within the ED.

To sustain these improvements, periodic reeducation sessions and routine reminders are planned beyond the initial intervention phase. Regular audits will be conducted to monitor continued adherence and identify further opportunities for optimization.

Second Cycle: Postintervention Evaluation

The second cycle, conducted from August 26, 2024, to December 26, 2024, assessed the effectiveness of the implemented interventions. The findings demonstrated substantial improvements, with 81.8% of patients meeting the 10-minute target for ECG acquisition and documentation. These results highlighted the success of the intervention strategies in streamlining ECG processing, ultimately enhancing patient care efficiency in the ED.

This structured clinical audit effectively identified existing gaps and implemented evidence-based interventions, leading to measurable improvements in ECG processing times within the ED.

Ethical considerations

Ethical approval for this study was obtained from the Kassala Teaching Hospital Ethics Committee. No identifiable information was collected. The study adhered to institutional guidelines and ethical principles for conducting clinical audits.

## Results

The findings of this study demonstrate significant improvements in both the timeliness and quality of ECG documentation from the first to the second audit cycle. These key changes are summarized in Table 2. Notably, the time from arrival to ECG acquisition reduced dramatically, with the proportion of patients receiving ECGs within the recommended 10-minute window increasing from 1 (4%) to 18 (81.8%). The p value for this change is less than 0.001, indicating a highly significant improvement. Additionally, the average time to ECG acquisition was reduced by 89.6%, reflecting a more efficient and streamlined process in the second audit cycle.

**Table 2:** Comparison of ECG process metrics between the first and second cycles The key ECG process metrics between the first and second audit cycles are compared. It highlights significant improvements in ECG acquisition time, documentation quality, and diagnostic accuracy, with areas needing further focus identified. Statistical tests used include chi-square (χ²) tests for categorical comparisons (such as proportions of documented elements and diagnosis frequencies) and an independent samples t-test for comparison of means (e.g., average time to ECG acquisition). A p value of less than 0.05 was considered statistically significant ECG: electrocardiogram; ACS: acute coronary syndrome

Parameter		First cycle	Second cycle	Change	Interpretation	Test statistic	p value
Time from arrival to ECG acquisition	≤10 minutes	1 (4%)	18 (81.8%)	77.8%	More patients received ECGs within the recommended 10-minute window	χ² = 22.78	<0.001
	Average time (minutes)	29.84	3.1	-89.6%	Significant reduction in time to ECG acquisition	-	-
Documentation quality	Rhythm documented	8 (32%)	20 (90.9%)	58.9%	Dramatic improvement in rhythm documentation	χ² = 8.89	0.003
	Rate documented	11 (44%)	22 (100%)	56%	All patients had heart rates documented in the second cycle	χ² = 6.86	0.009
	ST-segment documented	5 (20%)	22 (100%)	80%	Complete improvement in ST-segment documentation	χ² = 21.97	<0.00001
	PR interval documented	5 (20%)	3 (13.6%)	-6.4%	Minimal decline, indicating a need for further focus	χ² = 0.58	0.446
	QRS complex documented	6 (24%)	5 (22.7%)	-1.3%	Slight decline, suggesting room for improvement	χ² = 0.11	0.742
	Axis documented	5 (20%)	15 (68.2%)	48.2%	Significant improvement in axis documentation	χ² = 5.69	0.017
	No elements documented	13 (52%)	0 (0%)	-52%	Complete improvement; all cases had some level of documentation in the second cycle	-	-
Diagnostic findings	Normal ECG	1 (4.5%)	7 (28%)	23.5%	Increase in normal ECG findings, possibly due to better screening	χ² = 3.78	0.052
	ACS	12 (54.4%)	6 (24%)	-30.4%	Fewer ACS diagnoses, possibly reflecting improved diagnostic accuracy	χ² = 4.23	0.040
	Other causes (not ACS)	9 (40.9%)	12 (48%)	7.1%	Slight increase in non-ACS diagnoses	-	-

The quality of documentation also saw substantial improvements, particularly in the documentation of rhythm, rate, and ST-segment. The percentage of patients with rhythm documented increased by 58.9%, with a p value of 0.003, suggesting a statistically significant improvement. The documentation of the rate increased by 56%, with a p value of 0.009, indicating a significant enhancement. ST-segment documentation improved by 80%, with a p value less than 0.00001, reflecting a complete transformation in this area. However, the documentation of the PR interval and QRS complex showed only minimal changes, with p values of 0.446 and 0.742, respectively, suggesting that these areas still need attention. Importantly, no patients lacked documentation in the second cycle, marking a complete improvement in this regard.

In terms of diagnostic outcomes, the proportion of patients diagnosed with ACS decreased significantly from 54.4% to 24%, with a p value of 0.040, indicating that fewer ACS diagnoses were made in the second cycle, possibly reflecting improved diagnostic accuracy. In contrast, the proportion of patients diagnosed with other causes (non-ACS) increased slightly, from 40.9% to 48%, with no statistically significant change. The proportion of normal ECG findings also increased in the second cycle, from 4.5% to 28%, with a p value of 0.052, suggesting a marginally significant increase, possibly due to improved screening practices.

## Discussion

This clinical audit underscores the critical importance of prompt ECG acquisition and comprehensive documentation in managing patients with suspected ACS in Sudanese EDs. The marked improvements observed following targeted interventions align with prior research, reinforcing the efficacy of educational strategies and process optimization.

In the initial cycle, the average time from patient arrival to ECG acquisition was 29.84 minutes, with only one patient (4%) receiving ECG documentation within the recommended 10-minute window. Such delays can adversely impact patient outcomes, emphasizing the need for prompt action. Diercks et al. [2] reported that only about one-third of ACS patients received an ECG within 10 minutes of ED arrival, a challenge that our findings also reflect.

Following the implementation of targeted interventions, including interactive educational sessions, visual aids, and workflow adjustments, the second cycle demonstrated a dramatic reduction in ECG acquisition time to an average of 3.1 minutes. Moreover, 18 (81.8%) of patients met the 10-minute target for both ECG acquisition and documentation. This improvement is consistent with the findings of Chhabra et al. [5], who noted that interventions such as dedicated ECG technicians and focused triage education can significantly reduce door-to-ECG times.

The audit also revealed a noteworthy enhancement in the quality of ECG documentation. Initially, 13 (52%) of cases lacked documentation of essential ECG elements. After intervention, documentation rates for critical parameters, such as rhythm, rate, and ST-segment analysis, improved markedly, with compliance rates ranging from 20 (90.9%) to 22 (100%). These results align with studies that advocate for structured educational programs to enhance ECG interpretation and documentation skills among healthcare providers [6].

Furthermore, a quality improvement project in Saudi Arabia demonstrated that strategies, including human resource adjustments, technological enhancements, and improved interdepartmental collaboration, could significantly increase the proportion of patients receiving timely ECGs [7]. Similarly, the positive impact of interventions such as early patient notification, strategic placement of EKG machines, patient education, and modifications to triage protocols in reducing door-to-ECG times was highlighted [8].

Research by Zègre-Hemsey et al. [9] indicated that only 59% of patients with ischemic symptoms received an ECG within the recommended timeframe, with women experiencing longer delays than men. This gender disparity underscores the need for further targeted interventions to ensure equitable care.

The 2013 ACCF/AHA Guideline for the Management of ST-Elevation Myocardial Infarction by O’Gara et al. [10] emphasizes that standardizing protocols and continuous staff education are critical for achieving timely diagnosis and treatment in ACS management. Our findings support these recommendations, demonstrating that structured, protocol-driven educational interventions can lead to significant improvements in door-to-ECG times and documentation quality.

While the targeted interventions led to significant improvements in ECG acquisition times and overall documentation quality, it is essential to acknowledge that the documentation of certain ECG elements, such as the PR interval and QRS complex, experienced a decline following the intervention. This unexpected outcome may be attributed to the focused emphasis on parameters like rhythm, rate, and ST-segment analysis during training sessions, potentially leading to less attention on other components. Additionally, the increased workload or cognitive load on healthcare providers during rapid assessments might have contributed to this oversight. This finding underscores the necessity for comprehensive training programs that encompass all critical ECG elements and the implementation of robust documentation protocols to ensure uniform attention across all parameters.

Limitations

This audit has several limitations that should be considered when interpreting the findings. While we hypothesized that improved diagnostic accuracy and triage contributed to a higher number of normal ECGs and fewer ACS diagnoses in the second cycle, we lacked supporting data such as troponin levels, cardiologist reviews, or patient case-mix analysis. As such, these inferences must be interpreted cautiously. Other potential confounders, including symptom severity, time of day, and staff experience, were not controlled for. The absence of randomization or blinding introduces the risk of bias in documentation assessment, and the possibility of a Hawthorne effect, where staff alter their behavior due to awareness of being observed, cannot be ruled out. These methodological constraints are inherent to many clinical audits but must be transparently acknowledged. The sample size was relatively small, which may limit the generalizability of the results to other institutions or healthcare settings. The study was conducted in a single center, further narrowing the external validity. Additionally, while the audit demonstrated improvements in ECG timing and documentation following the interventions, it did not assess the long-term sustainability of these improvements or their direct impact on clinical outcomes such as door-to-balloon time, morbidity, or mortality. Future audits with larger sample sizes, longer follow-up periods, and multicenter participation are recommended to strengthen the evidence and assess the broader clinical implications of timely ECG processing.

## Conclusions

This audit demonstrated that targeted educational interventions significantly improved ECG acquisition times and documentation practices in suspected ACS cases at Kassala Teaching Hospital. While the impact on clinical outcomes remains unmeasured, the findings underscore the value of continuous training and performance monitoring in resource-limited emergency settings. Sustained improvement may be achieved through regular audits, standardized documentation, and simple technological aids. Focused workflow enhancements, rather than broad policy reforms, offer a practical path to improving ACS care.

## Appendices

**Table 3:** Audit parameters for ECG acquisition, interpretation, and documentation in emergency departments The key parameters used to assess the efficiency and documentation quality of ECG processes in the ED are outlined. It includes time intervals for different stages of ECG handling, the rationale for ECG performance, and the quality of documentation ECG: electrocardiogram; ED: emergency department Source: [1]

No.	Parameters
1	Time from arrival to ECG acquisition
2	Time from ECG acquisition to interpretation
3	Time from interpretation to documentation
4	Indication for ECG (typical chest pain vs. atypical presentation)
5	Quality of ECG documentation (rate, rhythm, axis, ST segment, QRS complex, and PR interval)
